# Supplementary material for: Transfer learning for biomedical named entity recognition with neural networks
Source: Bioinformatics. 2018 Jun 1;34(23):4087–94. doi: 10.1093/bioinformatics/bty449 (PMC6247938; doi:10.1093/bioinformatics/bty449)
Supplement: Supplementary Data [file bty449_supp.zip › bty449-suppl_data/appendix_A1.pdf]

Appendix A

We have provided precision, recall, and F1-scores of each model (baseline and transfer learning) for each corpus in Table A1. We also include the scores reported by Habibi *et al.* (2017) for comparison. We measure statistical significance using a two-tailed t-test.

Table 3. Performance values in terms of precision, recall and F1-score for our baseline (B) and transfer learning (TL) methods and those reported by Habibi et al. (2017) (H). Baseline values are derived from training on the target dataset only, while transfer learning values are derived by training on the source dataset followed by training on the target dataset. The baseline and transfer learning methods were trained for five runs, and the results were averaged. Scores from Habibi et al. (2017) are reported directly. Bold: best scores, *: significantly worse than the TL model ($p \leq 0.05$), **: significantly worse than the TL model ($p \leq 0.01$).

			Precision (%)			Recall (%)			F1-score (%)		
Entity type	Corpus	No. of annotations	B	H	TL	B	H	TL	B	H	TL
Chemicals	BioSemantics	386110	**85.68**	80.88**	85.03	88.82	83.14**	**88.97**	**87.20**	81.99**	86.95
	CDR	15915	92.45	92.57	**92.70**	89.95*	88.77**	**90.62**	91.18*	90.63**	**91.64**
	CHEMDNER patent	65685	83.15	83.33	**83.43**	88.79	85.45**	**88.83**	85.87	85.38**	**86.05**
Diseases	Arizona Disease	3425	75.68	**76.64**	76.10	75.67	72.47	**77.07**	75.65*	74.49*	**77.48**
	CDR	12617	82.07	**84.19**	82.03	82.12	**82.79**	82.63	82.09	**83.49**	82.32
	miRNA	2159	76.22	**80.86**	77.95	78.99	75.26**	**80.21**	77.57	77.96*	**79.05**
	NCBI Disease	6881	81.26	**85.31**	82.62	86.50	83.58**	**86.95**	83.79	84.44	**84.72**
	Variome	5904	86.83*	84.51**	**88.33**	82.37**	**87.64**	85.45	84.53**	86.05**	**86.86**
Species	CellFinder	479	77.97	79.03	**80.20**	85.52*	**92.45**	91.64	81.51*	85.22*	**85.45**
	Linneaus	4259	92.74	**93.57**	92.80	90.38**	93.24	**94.29**	91.49**	93.40	**93.54**
	LocText	276	96.00*	91.00**	**98.66**	88.16**	89.39**	**95.53**	91.91**	89.50*	**97.06**
	miRNA	722	**91.63**	87.88	91.51	94.84	92.95**	**95.98**	93.20	90.34**	**93.68**
	S800	3708	**75.22**	74.55	74.34	74.40	69.81**	**75.96**	74.77	72.10**	**74.98**
	Variome	182	**71.53**	59.00**	69.63	73.33**	**89.39**	87.33	72.32**	71.08**	**77.32**
Genes/proteins	BioCreative II GM	24596	**79.24**	77.50*	78.62	77.86*	78.13**	**78.71**	78.54	77.82	**78.66**
	BioInfer	4378	92.77	92.64**	**93.59**	90.20*	89.59**	**91.95**	91.46*	91.09**	**92.76**
	CellFinder	1750	88.00	88.73	**91.83**	64.21**	65.07**	**77.34**	74.16**	75.08**	**83.85**
	DECA	6324	67.52	**75.27**	68.11	**76.03**	66.16**	75.42	71.46	70.42**	**71.53**
	FSU-PRGE	59489	85.44	**87.26**	85.63	86.54	**87.24**	86.51	85.98	**87.25**	86.06
	IEPA	1110	86.32	**88.01**	87.40	87.92	85.67**	**89.93**	87.10	86.82**	**88.64**
	LocText	1395	72.22*	–	**75.55**	75.97	–	**76.88**	74.03**	–	**76.16**
	miRNA	1058	76.69	75.95*	**79.64**	77.28	78.09	**78.58**	76.92*	77.00**	**78.98**
	Variome	4617	**90.60**	87.47	90.05	91.66	**96.87**	92.35	91.12	**91.93**	91.18
