# Supplementary material for: Transfer learning for biomedical named entity recognition with neural networks
Source: Bioinformatics. 2018 Jun 1;34(23):4087–94. doi: 10.1093/bioinformatics/bty449 (PMC6247938; doi:10.1093/bioinformatics/bty449)
Supplement: Supplementary Data [file bty449_supp.zip › bty449-suppl_data/additional_file_1.pdf]

Supplementary Information: Transfer learning for biomedical named entity recognition with neural networks

John Giorgi, Gary D. Bader

This document contains supplementary information for the paper *Transfer learning for biomedical named entity recognition with neural networks.*

1 Importance of each layer

In order to determine the importance of each layer in the long short term memory network-conditional random field (LSTM-CRF), we cumulatively transferred each layer (starting from the lowest layer) learned on the source silver-standard corpora (SSCs) for learning on four select gold-standard corpora (GSCs) — one for each entity class.

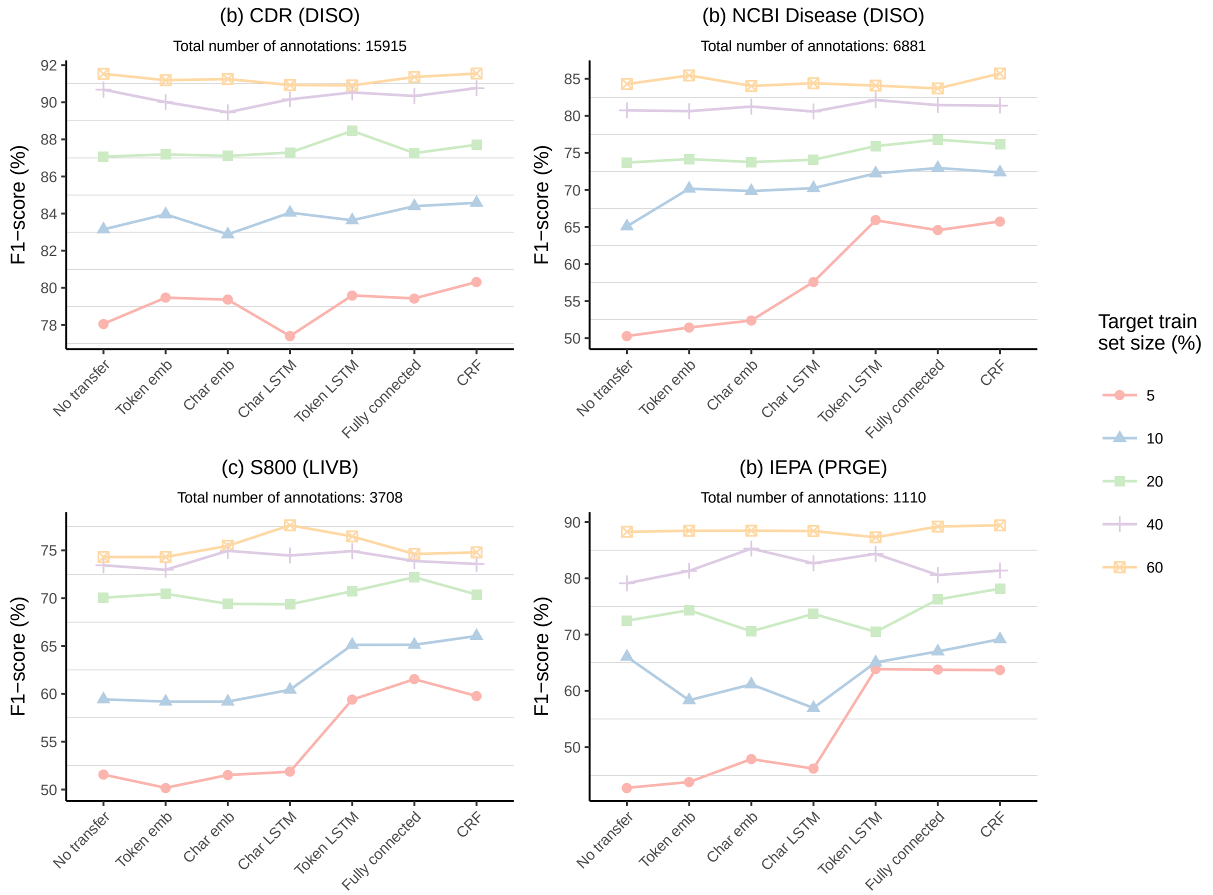

Figure 1: Impact of transferring the parameters up to each layer of the LSTM-CRF model using various train set sizes on the target dataset. Baseline (No transfer) corresponds to training the model only with the target data set, and transfer learning corresponds to training on the source data set followed by training on the target data set. The number of training examples used as the target training set is reported as a percent of the overall GSC size (e.g., for a GSC of 100 documents, a target train set size of 60% corresponds to 60 documents).

2 Blacklists for silver-standard corpora

In an effort to reduce noise in the SSCs, a selection of entities present but not annotated in any of the GSCs of the same entity type were removed from the SSCs. For example, certain text spans such as 'genes', 'proteins', and 'animals' are annotated in the SSCs but not annotated in any of the GSCs of the same entity type, and so were removed from the SSCs. Blacklists are available in **additional_file_2.zip**. Alternatively, they are avalible at https://github.com/BaderLab/Transfer-Learning-BNER-Bioinformatics-2018/.
